# Familial TLR8 gain-of-function presenting as HLH with response to IFN-γ blockade

**DOI:** 10.70962/jhi.20260101

**Published:** 2026-11-10

**Authors:** John S. Barber, Nermina Saucier, Megan Murphy, Elizabeth G. Schmitz, Kristen McDaniels, Cathleen Cook, Guglielmo Venturi, John W. Sleasman, Kris M. Mahadeo, Megan A. Cooper, Talal Mousallem

**Affiliations:** 1 Division of Pediatric Allergy and Immunology, Department of Pediatrics, Duke University School of Medicine, Durham, NC, USA; 2 Division of Rheumatology & Immunology, Department of Pediatrics, Washington University School of Medicine, St. Louis, MO, USA; 3 Pediatric Hematology and Oncology, Carilion Roanoke Memorial Hospital, Roanoke, VA, USA; 4 Department of Pediatrics, Pediatric Transplant and Cellular Therapy, Duke University School of Medicine, Durham, NC, USA

## Abstract

Gain-of-function variants in TLR8 cause X-linked immune dysregulation characterized by cytopenias, bone marrow failure, and lymphoproliferation. We report an 8-year-old male in whom TLR8-GOF manifested as hemophagocytic lymphohistiocytosis, an association not previously described. Treatment with emapalumab was associated with clinical improvement.

## Teaser

Barber et al. report a novel TLR8 gain-of-function variant presenting as hemophagocytic lymphohistiocytosis. Clinical response to IFN-γ blockade with emapalumab highlights targeted cytokine inhibition as a potential adjunctive therapy prior to transplantation. The mother harbored the variant and had autoimmune cytopenia, expanding the phenotypic spectrum of this condition in affected females.

**Figure 1.**
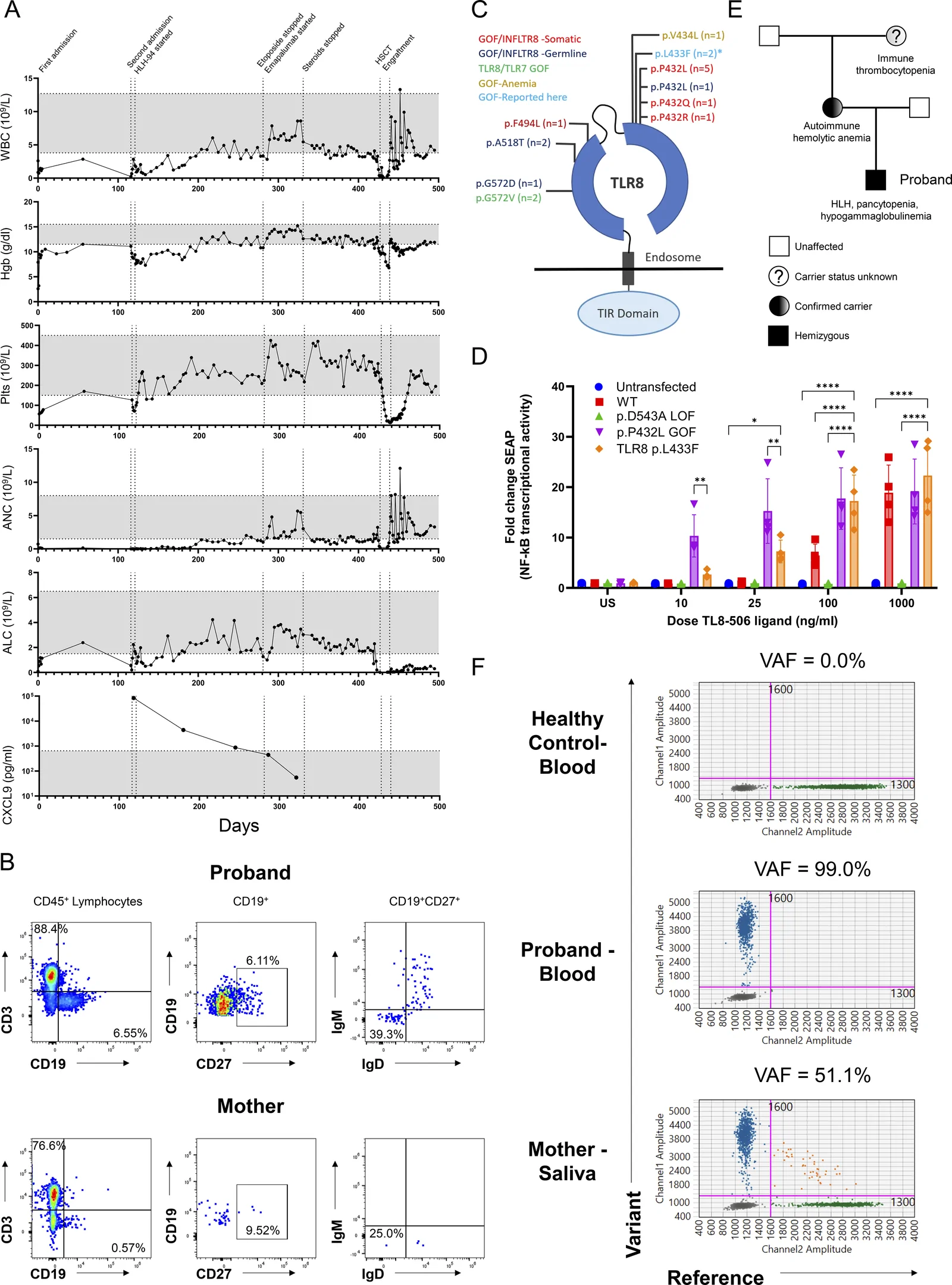
**Laboratory, genetic and functional characterization of the p.Leu433Phe TLR8 variant. (A)** Serial laboratory values over the clinical course, including white blood cell count (WBC), hemoglobin (Hgb), platelets (Plt), ANC, absolute lymphocyte count (ALC), and CXCL9. Vertical dashed lines denote key clinical time points. Shaded regions indicate normal reference ranges. **(B)** Flow cytometric immunophenotyping of peripheral blood cells from the proband (top) and mother (bottom). Cells previously gated on lymphocytes and CD45^+^. **(C)** Schematic of the TLR8 protein illustrating the locations of previously reported GOF variants and the novel variant identified in the proband and mother. **(D)** Functional assessment of the p.Leu433Phe variant compared with WT, a loss-of-function control (p.D543A LOF), and a known GOF variant (p.P432L GOF). NF-κB reporter activity is shown across a dose range of TL8-506 ligand (ng/ml). Reporter cells were transiently transfected with 500 ng of WT or variant TLR8 plasmid using Lipofectamine (Thermo Fisher Scientific), and 48 h after transfection, cells were stimulated with TL8-506 (InvivoGen) for 18 h. NF-κB transcriptional activity was measured by quantifying secreted alkaline phosphatase by the QUANTI-BLUE assay (InvivoGen). Fold change in secreted alkaline phosphatase (SEAP) activity was calculated by normalizing data to untransfected wells without addition of TL8-506. Data are presented as mean ± SD of biological replicates. Data were generated in four independent experiments. Statistical analysis was performed using ordinary two-way ANOVA followed by a multiple-comparisons test to compare variant responses at each agonist concentration. For clarity, only statistical comparisons involving the TLR8 p.L433F variant are shown. *P < 0.05; **P < 0.01; ***P < 0.001; ****P < 0.0001. **(E)** Pedigree of the family. **(F)** ddPCR VAF in healthy control blood, proband blood, and mother saliva. This was performed once.

An 8-year-old male with a history of recurrent oral ulcers presented with high fevers, fatigue, anorexia, and cough. Initial evaluation revealed pancytopenia with white blood cell count 0.75 × 10^3^/μl (absolute neutrophil count [ANC] 100/μl), hemoglobin 3.8 g/dl, and platelets 64 × 10^9^/liter (Fig. 1 A). C-reactive protein was markedly elevated at 71.1 mg/liter. Respiratory PCR was positive for respiratory syncytial virus (RSV) and *Mycoplasma pneumoniae*. Bone marrow biopsy demonstrated trilineage hematopoiesis without malignancy but with prominent T cell and histiocyte infiltration, including rare hemophagocytic forms. He was initially treated with supportive care and broad-spectrum antibiotics with resolution of fever and was discharged with presumptive diagnosis of infection-related myelosuppression.

Five months later, after complete resolution of his initial respiratory illness, he developed recurrent fevers, an eyelid stye, oral ulcers, and maculopapular rash. Pancytopenia had recurred with an ANC of 0/μl. Extended respiratory viral PCR testing on a nasal swab, including RSV, was negative. Peripheral blood viral PCRs (including HHV-6, CMV, and EBV) and parvovirus serologies were also negative. Immunologic studies demonstrated lymphopenia with CD3^+^ T cells 500/μl (CD4^+^ 230/μl, CD8^+^ 250/μl), marked B cell lymphopenia at 30/μl, and natural killer (NK) cell lymphopenia at 10/μl. IgG (1,027 mg/dl) and IgA (191 mg/dl) were normal, with only a mildly reduced IgM of 34 mg/dl. Laboratory evaluation also demonstrated ferritin of 4,021 ng/ml, soluble IL-2 receptor of 15,628 U/ml, CXCL9 of 83,818 pg/ml, mild hypertriglyceridemia (189 mg/dl), and progressive hypofibrinogenemia, with fibrinogen declining to 139 mg/dl prior to treatment. Abdominal imaging demonstrated mild hepatosplenomegaly.

During this second episode, he met seven of eight hemophagocytic lymphohistiocytosis (HLH)-2004 diagnostic criteria for HLH and was initiated on a modified HLH-94 protocol with dexamethasone and etoposide. Despite clinical improvement, he had persistent leukopenia, anemia, and elevated inflammatory markers. Immunoglobulin levels declined progressively, with nadirs of IgG 357 mg/dl, IgM 20 mg/dl, and IgA 36 mg/dl, necessitating immunoglobulin replacement therapy. While on treatment, extended immunophenotyping demonstrated an inverted CD4:CD8 ratio and reduced total and memory B cells, with a preserved proportion of class-switched memory B cells (Fig. 1 B). Given incomplete hematologic recovery and concern for cumulative toxicity, therapy was transitioned to IFN-γ blockade with emapalumab. Following this transition, further improvement in cytopenias and inflammatory markers was observed (Fig. 1 A).

Targeted genetic panels for primary HLH and bone marrow failure syndromes were unrevealing. Proband-only whole-genome sequencing identified a hemizygous variant in TLR8 (NM_138636.5:c.1299G>C, p.Leu433Phe), adjacent to residues at which gain-of-function (GOF) variants have been recurrently identified (Fig. 1 C). The patient and his parents were consented and enrolled in institutional review board–approved research studies at the author institutions. The variant is absent from the population database gnomAD.

Although standard in silico prediction algorithms (including combined annotation dependent depletion [CADD] and AlphaMissense) predicted the variant to be benign, functional studies confirmed GOF activity, though of lesser magnitude than most previously reported variants, consistent with INFLTR8 (inflammation, neutropenia, bone marrow failure, and lymphoproliferation) syndrome due to GOF in TLR8 (TLR8-GOF) (Fig. 1 D) (1, 2). The same variant was identified in his mother, whose history was notable for recurrent autoimmune hemolytic anemia requiring corticosteroids and rituximab. Despite >5 years having elapsed since her last rituximab dose, immunophenotyping demonstrated markedly reduced total B cells (Fig. 1 B). Germline status of the variant in the mother was confirmed by droplet digital PCR (ddPCR), which demonstrated a variant allele fraction (VAF) of ∼50% and a VAF of 100% in the child (Fig. 1 F). The maternal grandmother had immune thrombocytopenia, though her carrier status is unknown (Fig. 1 E).

Following the diagnosis of TLR8-GOF, he underwent matched (10/10 HLA) unrelated allogeneic hematopoietic stem cell transplantation (HSCT) using a bone marrow graft from a CMV-seronegative, blood type O+ donor (recipient blood type A+) following conditioning with fludarabine, busulfan, and (rabbit) antithymocyte globulin. Neutrophil engraftment occurred on day +11 (Fig. 1 A) and platelet engraftment on day +23. Early chimerism studies demonstrated >98% donor chimerism in whole blood and myeloid fractions and 55% in the T cell fraction. At day +153, he is transfusion and growth factor independent and remains clinically well. He did not experience any major post-HSCT complications such as sinusoidal obstructive syndrome, infection, and/or graft-versus-host disease.

TLR8-GOF is a recently described X-linked dominant inborn error of immunity characterized by multilineage cytopenias, including severe neutropenia, lymphoproliferation, and susceptibility to infection due to mosaic or germline missense variants in *TLR8*. The disorder arises from enhanced TLR8 signaling in myeloid cells, driving increased NF-κB activation and inflammatory cytokine production. Despite this innate immune overactivation, the clinical phenotype is dominated by bone marrow failure and immune dysregulation, likely mediated through cell-extrinsic effects on adaptive immune compartments. This patient’s presentation is consistent with prior reports (1, 2).

This report expands the literature of TLR8-GOF in several important ways. This represents the first presentation of TLR8-GOF as HLH. While prior reports have described inflammatory cytokine elevation, HLH has not been a defining manifestation. The patient met HLH-2004 diagnostic criteria, suggesting that dysregulated TLR8 signaling can drive sufficient immune activation to produce HLH. We view HLH in this case as the hyperinflammatory manifestation of underlying TLR8-GOF rather than a competing diagnosis, given the substantial overlap between HLH-2004 criteria and features intrinsic to TLR8-GOF itself. Importantly, this patient achieved meaningful clinical improvement with HLH-directed therapy, in contrast to previously reported TLR8-GOF patients who had poor responses to less aggressive immunosuppressive approaches (2). These findings have important diagnostic and therapeutic implications: TLR8-GOF should be considered in patients with presumed primary HLH and persistent cytopenias when standard genetic evaluation is unrevealing, and those with confirmed TLR8-GOF may require aggressive immunosuppression.

The novel identified variant (c.1299G>C; p.Leu433Phe) is located adjacent to multiple pathogenic GOF variants and confers a less pronounced in vitro GOF phenotype relative to previously reported TLR8-GOF variants. This may explain this patient’s later presentation compared with previously reported germline cases that manifested in infancy and raises the possibility that variant-specific signaling strength contributes to clinical heterogeneity. Despite demonstrating GOF activity in vitro, p.Leu433Phe was predicted to be benign by standard in silico algorithms, underscoring the importance of functional validation when evaluating novel TLR8 variants. Further characterization of the variant, including assessment of TLR8 protein expression in primary cells, was limited by sample availability. Although formal diagnosis of HLH was established after resolution of the initial infections with RSV and *M. pneumoniae*, his presentation at the time of infection was also accompanied by marked cytopenias and systemic inflammation, suggesting that infection may have precipitated or unmasked underlying immune dysregulation. Given TLR8’s role in sensing microbial single-stranded RNA (ssRNA) and driving downstream inflammatory signaling, such exposures may reveal otherwise latent immune dysregulation.

The family history also expands the phenotype of TLR8-GOF in affected females. The patient’s mother carried the variant and had a history of autoimmune hemolytic anemia, and the maternal grandmother had immune thrombocytopenia, though her carrier status is unknown. Both presentations differ markedly from the INFLTR8 syndrome; other previously reported females with germline TLR8-GOF had more prominent hematologic manifestations with pure red cell aplasia (2, 3). Autoimmune cytopenias are not a prominent feature of TLR8-GOF; the only clear description of autoimmune cytopenias in the context of TLR8 human disease comes from monozygotic twins with a GOF variant complicated by increased protein degradation and overactivation of the TLR7 pathway (4). Furthermore, TLR8 escapes X-chromosome inactivation in human immune cells (5), suggesting cells in heterozygous females may co-express both WT and mutant TLR8, rather than expressing a single allele as in conventional X-linked disorders. The implications of this biology for disease expression remain incompletely understood.

This case also highlights the potential role of IFN-γ blockade in TLR8-GOF, which can be mechanistically rationalized in the context of TLR8 biology. Activation of TLR8 via ssRNA sensing in monocytes and macrophages drives NF-κB–dependent transcription of proinflammatory cytokines that promote T helper 1 polarization. Sustained activation of this pathway can amplify downstream IFN-γ production by T cells and NK cells, creating a feed-forward loop of macrophage activation and cytokine release. This cytokine milieu closely mirrors that observed in HLH, where IFN-γ is a central pathogenic driver of immune-mediated tissue injury and cytopenias. Within this framework, blockade of IFN-γ with emapalumab interrupts the aberrant downstream inflammatory cascade. This patient’s markedly elevated CXCL9, a surrogate marker of IFN-γ activity, provided direct rationale for this approach. The clinical response observed here following initiation of emapalumab supports further investigation of IFN-γ blockade as a complementary strategy to established HLH-directed protocols in patients with TLR8-GOF. These observations also provide a mechanistic rationale for further investigation of JAK inhibition, which attenuates signaling downstream of IFN-γ and other proinflammatory cytokines, as an additional therapeutic approach. More broadly, this case adds to a growing body of evidence that enhanced activation of innate immune signaling pathways, independent of a primary lymphocyte-driven mechanism, can be sufficient to precipitate HLH.

Allogeneic HSCT remains the only curative therapy for primary HLH, and in this patient, it resulted in resolution of cytopenias and immune dysregulation.

## Materials and methods

### ddPCR

Genomic DNA (gDNA) was isolated from the indicated patient samples or a healthy individual, and custom primer/probes specific for the patient’s missense variant and reference alleles were conjugated to Fluorescein amidite (FAM) and Hexachlorofluorescein (HEX) fluorophores, respectively (BIO-RAD #10049047). ddPCR reactions were performed as previously described (2).

### Transfection studies in TLR8-deficient NF-κB reporter cell line

Variant (c.1299G>C; p.Leu433Phe) was tested using HEK-Blue Null1 NF-κB reporter cells (InvivoGen) lacking TLR8 expression as previously described (1).

### Flow cytometry

Processing and immunophenotyping of peripheral blood mononuclear cells were performed using standard flow cytometry assays.

## Data Availability

The data underlying this study are included in the published article. The *TLR8* variant Leu433Phewas deposited in ClinVar (Variation ID: 4886965).
